# Case report: Fabrication of a dental implant guide based on tetrahedron positioning technology

**DOI:** 10.1186/s12903-021-01694-x

**Published:** 2021-07-07

**Authors:** Jie Lin, Zhenxiang Lin, Zhiqiang Zheng

**Affiliations:** 1 Fujian Key Laboratory of Oral Diseases, School and Hospital of Stomatology, Fujian Medical University, 246 Yangqiao Zhong Road, Fuzhou, 350002 Fujian People’s Republic of China; 2 Department of Crown and Bridge, School of Life Dentistry At Tokyo, The Nippon Dental University, 1-9-20 Fujimi, Chiyoda-ku, Tokyo, 102-8159 Japan; 3 Department of Stomatology, Hospital of Fujian Provincial Authorities, 68 Guping Road, Fuzhou, 350001 Fujian People’s Republic of China

**Keywords:** Computer-assisted therapy, Tetrahedron positioning technology, Implant surgical guides, Cone-beam computed tomography

## Abstract

**Background:**

Conventional static computer-assisted implant surgery (s-CAIS) requires special equipment, such as 3D printers or computer numerical control (CNC) lathes. We present a low-cost workflow for manufacturing dental implant guides based on tetrahedron positioning technology (TPT). The aim of this case report was to use a surgical guide technique for dental implant placement using tetrahedron positioning technology.

**Case presentation:**

A 28-year-old man consulted for the treatment of a missing right first mandibular molar by implant placement. The cone-beam computed tomography (CBCT) data were imported into medical image processing software for analysis, and the implant design was simulated. The implant design on CBCT was transferred to the mandibular model using TPT, and the implant surgical guide was made to guide the dental implant operation. CBCT was performed postoperatively and compared with the preoperative design to check the accuracy. The central deviation of the implant head was 0.31 mm, the central deviation of the implant apex was 0.93 mm, and the implant angular deviation was 2.45°.

**Conclusion:**

The use of tetrahedral positioning technology based on CBCT data is a new method for making implant guides. It is a promising technique offering a highly predictable outcome and lower risk of iatrogenic damage. However, these results should be interpreted with care since they are based on limited evidence from a case report. Larger population studies with longer follow-up periods and standardized experimental studies are required.

## Background

In recent years, dental implant surgery based on cone-beam computed tomography (CBCT) and three-dimensional (3D) printing surgical guide technology has been widely used in clinical practice and plays an important role in improving the accuracy of implantation and final restoration [1–3]. Any virtual implant planning system using a 3D software application in combination with implant placement by means of a computer-aided design/computer-aided manufacturing (CAD/CAM)-processed surgical guide is defined as a system for static computer-assisted implant surgery (s-CAIS) [4]. Although s-CAIS seems to offer a beneficial treatment option in terms of accuracy [5] and operator experience [6], the economic effects in terms of time efficiency and treatment costs are unclear [7]. For s-CAIS, 3D-printable surgical implant guides are most widely used [8, 9], whereas the production of these implant guides by additive or subtractive manufacturing technologies is so-called conventional s-CAIS. These conventional s-CAISs require special equipment, such as 3D printers or computer numerical control (CNC) lathes. Undoubtedly, most implantation procedures for a single missing tooth are performed freehand. Nonetheless, the high processing cost of the surgical guide, which can be almost equal to the cost of the implant in some places of the world, hinders dentists from using surgical guides. This results in inaccurate positioning of the implant and a higher risk of misplacing the implant. Therefore, a low-cost method is needed as an alternative to conventional s-CAIS so that more dentists can benefit from digital technology to place dental implants.

Tetrahedron positioning technology (TPT) is a method for making implant surgical guides oriented by coordinates. It can transfer the guide design from the computer to the dental stone model through simple equipment and realize the low-cost production of implant guides. In brief, the polygon on the plane has at least three sides, the geometry of the space has at least four faces, and the tetrahedron is the simplest geometry of the space. Given the position of two points on the plane and the distance between the two points and a third point, the position of the third point can be located; given the position of three points in the tetrahedron and the distance between the three points and a fourth point, the position of the fourth point can be located. This can be mathematically proven by the formula for the distance between two points in 3D space [10].

**Fig. 1:**
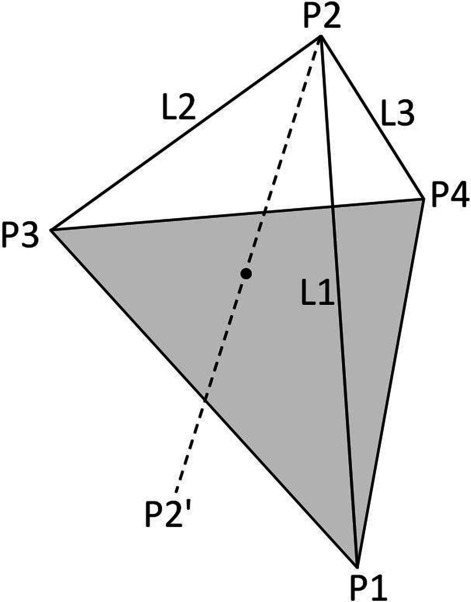
Schematic diagram of tetrahedron positioning technology. Given the position of three points (P1, P3 and P4) in the tetrahedron and the distance (L1, L2 and L3) between the three points and a fourth point (P2), the position of the fourth point can be located

Consider the three points P1, P3 and P4 with known coordinates of (x1, y1, z1), (x3, y3, z3) and (x4, y4, z4), respectively, as shown in Fig. 1. Then, the known distances from P2 (x2, y2, z2) with unknown coordinates to the three points are L1, L2 and L3, respectively, and the coordinates of point P2 can be calculated by:

$$\left( {{\text{x}}2 - {\text{x}}1} \right)^{2} + \left( {{\text{y}}2 - {\text{y}}1} \right)^{2} + \left( {{\text{z}}2 - {\text{z}}1} \right)^{2} = {\text{L}}1^{2}$$

$$\left( {{\text{x}}2 - {\text{x}}3} \right)^{2} + \left( {{\text{y}}2 - {\text{y}}3} \right)^{2} + \left( {{\text{z}}2 - {\text{z}}3} \right)^{2} = {\text{L}}2^{2}$$

$$\left( {{\text{x}}2 - {\text{x}}4} \right)^{2} + \left( {{\text{y}}2 - {\text{y}}4} \right)^{2} + \left( {{\text{z}}2 - {\text{z}}4} \right)^{2} = {\text{L}}3^{2}$$

This is a system of quadratic equations with three variables, and there are only two solutions for the coordinates (viz. P2 and P2′), which are two points symmetrical to the ABC plane. In dental practice, it is clear that point P2 needs to be above the occlusal plane. This is the basis of TPT in the application of implant guides.

This case report presents a novel method to make dental implant guides and demonstrates the usefulness of TPT for creating implant guides. The aim of this case report was to use a surgical guide technique for dental implantation using TPT.

## Case presentation

### Outline of the case

A 28-year-old patient was referred to the Department of VIP Dental Service, School and Hospital of Stomatology, Fujian Medical University, with a missing right first mandibular molar (tooth 46). The main complaint was the loss of right lower posterior tooth for 4 months. The patient's right lower posterior tooth was extracted 4 months prior due to "cavities". The patient had no history of cardiovascular disease, diabetes, infectious diseases or allergies. Tooth 46 was absent, the occlusal-gingival distance was 7 mm, there was no obvious absorption in the alveolar crest, the adjacent teeth were not loose, and the mandibular right second molar was mesialized.

**Fig. 2:**
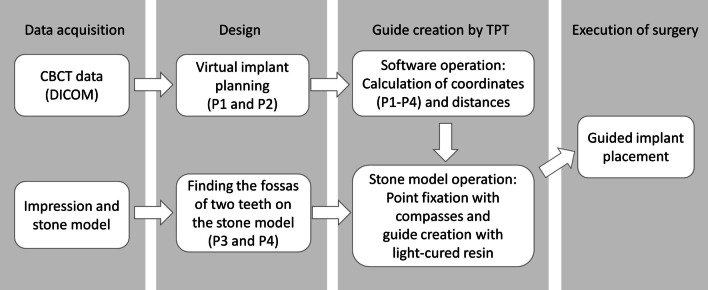
Workflow diagram for manufacturing a guide by tetrahedron positioning technology

The workflow diagram followed the sequence shown in Fig. 2. The treatment plan involved a single implant treatment guided by an implant surgical guide for tooth 46 to support the fixed restoration design. The steps were as follows: (1) CBCT (iCAT, KaVo, Germany) was performed, and a mandibular impression was made; (2) the CBCT data were imported into medical image processing software (Mimics 10.0, Materialise, Belgium), and the implant design was simulated; (3) the implant design on CBCT was transferred to the mandibular model using TPT, and the implant surgical guide was made to guide the dental implant operation.

The research protocol was reviewed and approved by the Research Ethics Committee at the School and Hospital of Stomatology, Fujian Medical University (No. 2020-CX-32).

### Materials for TPT-based implant guide

**Fig. 3:**
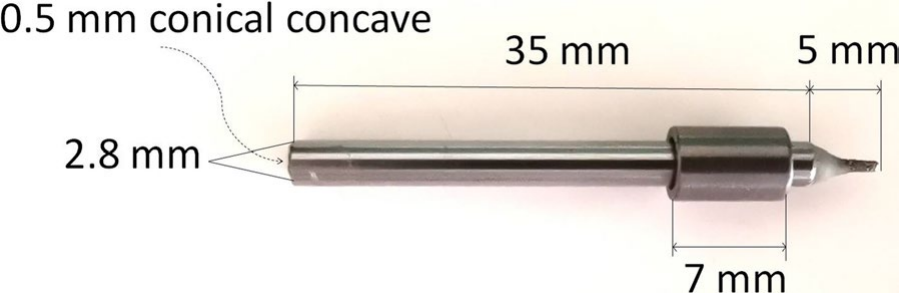
Design of the cylindrical guide bar and titanium guide ring (inner diameter, 2.85 mm)

The materials used were as follows: (1) a cylindrical bar (Hengrun, China) with a diameter of 2.80 mm and a length of 35.00 mm, extending 5.00 mm to a tip from the centre of one end and ground to a shallow concave depth of 0.50 mm at the centre point of the other end; a home-made titanium guide ring with an outer diameter of 5.00 mm, a length of 7.00 mm, and an inner diameter of 2.85 mm (Fig. 3); (2) two compasses (H2030, Hero, China); (3) electronic digital callipers (ARTPOL, Jiangsu Jingjiang, China) with an accuracy of 0.01 mm; and (4) light-cured temporary crown resin (Revotek LC, GC, Japan).

### CBCT and virtual implant planning

**Fig. 4:**
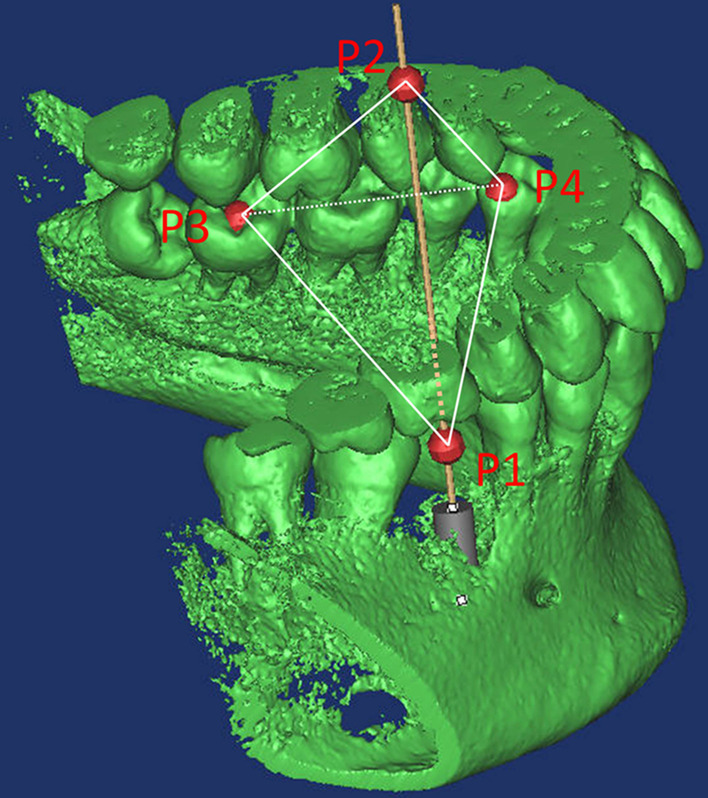
Virtual implant model in software for the application of tetrahedron positioning technology

CBCT was used to scan the mandibular dentition and alveolar bone of the patient. CBCT was performed at a voltage of 120 kV, scan current of 5.00 mA, exposure time of 7.0 s, resolution of 0.2 mm, image matrix size of 800 × 800 pixels, and axis layer thickness of 0.2 mm. A total of 304 two-dimensional tomography images were obtained. The data were saved in digital imaging and communications in medicine (DICOM) format and imported into Mimics 10.0 for processing to establish a three-dimensional morphological model including the tooth and mandibular alveolar bone. A cylindrical structure was established to simulate the implant diameter (4.1 mm), length (12 mm), position and orientation (Fig. 4).

### TPT-based guide production

Production of the TPT-based guide consisted of two main steps: software operation and dental stone model operation. The goal was to transfer the important design in the software to the dental stone model through TPT.

The first step was performed in Mimics 10.0 software. The diameter of the cylindrical implant was reduced to 0.1 mm to accurately locate its junction with the gingival mucosa, which was designated as point P1 (Fig. 4). The position of point P2 was determined by extending the cylindrical implant from P1 to the occlusal side by 39 mm. The central fossa of tooth 37 and the distal fossa of tooth 34 were designated as P3 and P4.

The three-dimensional coordinate values of the four points read by Mimics 10.0 software were as follows: P1 (60.06, 65.50, 46.95); P2 (70.77, 57.90, 83.63); P3 (105.50, 76.68, 49.56); and P4 (97.70, 50.62, 47.73). According to the formula for the distance between two points [8], if the coordinates of two points are $A(x_{1} ,y_{1} ,z_{1} )$ and $B(x_{2} ,y_{2} ,z_{2} )$, then

$$|AB| = \sqrt {(x_{1} - x_{2} )^{2} + (y_{1} - y_{2} )^{2} + (z_{1} - z_{2} )^{2} }$$

where P1–P3 = 46.86 mm, P1–P4 = 40.47 mm, P2–P3 = 52.15 mm, and P2–P4 = 45.46 mm.

**Fig. 5:**
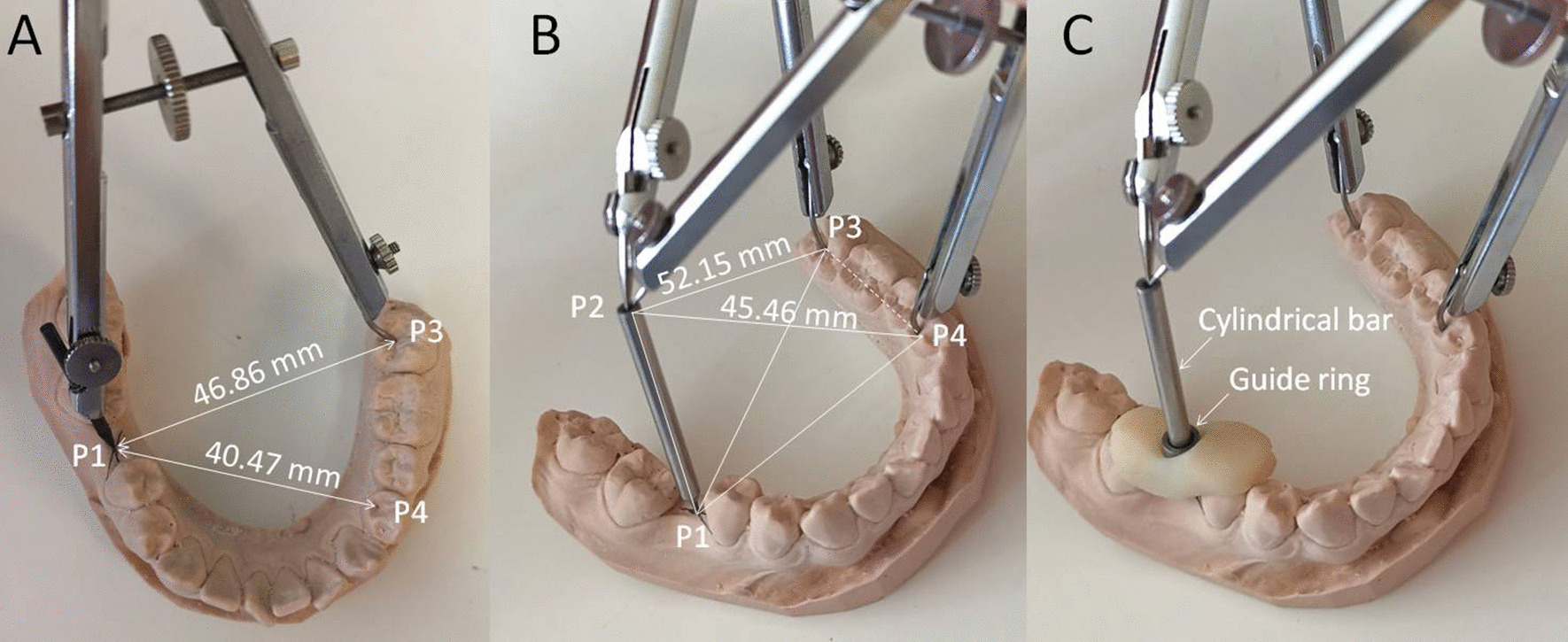
Stone model operation. **A** Location of the implantation site P1. **B** Connection of the four points to form a stable tetrahedron, location of P2 through P3 and P4, and determination of the distance between P1–P2 as 39 mm. **C** Placement of the guide ring followed by fixation with a light-cured temporary crown resin

The second step was performed on the dental stone model. A mandibular impression was taken with an alginate impression material and poured into a stone cast. A compass was used on the dental stone model with the central fossa of tooth 37 (P3) and the distal fossa of tooth 34 (P4) as the centre and 46.86 mm (P1–P3 distance) and 40.47 mm (P1–P4 distance) as the radius. Two arcs were drawn on the model of missing tooth 46, and the intersection point was the corresponding position of P1 on the model (Fig. 5A). At the P1 position of the model, a shallow recess with a depth of 0.5 mm was ground. The tip of the cylindrical guide bar was placed in the shallow recess, and the other end was fixed with P3 and P4 using two compasses (Fig. 5B). The length of the two compasses was set to 52.15 mm and 45.46 mm, respectively, to locate the implantation site and direction of the implant. A titanium guide ring with an inner diameter of 2.85 mm was sleeved into the cylindrical bar. A partial-arch tooth-supported implant surgical guide was designed for the missing right first mandibular molar. The surgical template was designed with a thickness of 2–3 mm. The light-cured temporary crown resin was fixed to the adjacent teeth, and the light-cured guide was completed (Fig. 5C). The distance from the upper edge of the guide ring to P1 was 10.69 mm, the thickness of the mucosa was 4.00 mm, and the implantation depth was 12.00 mm. The guiding depth is equal to the sum of these three lengths: 10.69 mm + 4 mm + 12 mm = 26.69 mm.

### Guide implantation and accuracy

**Fig. 6:**
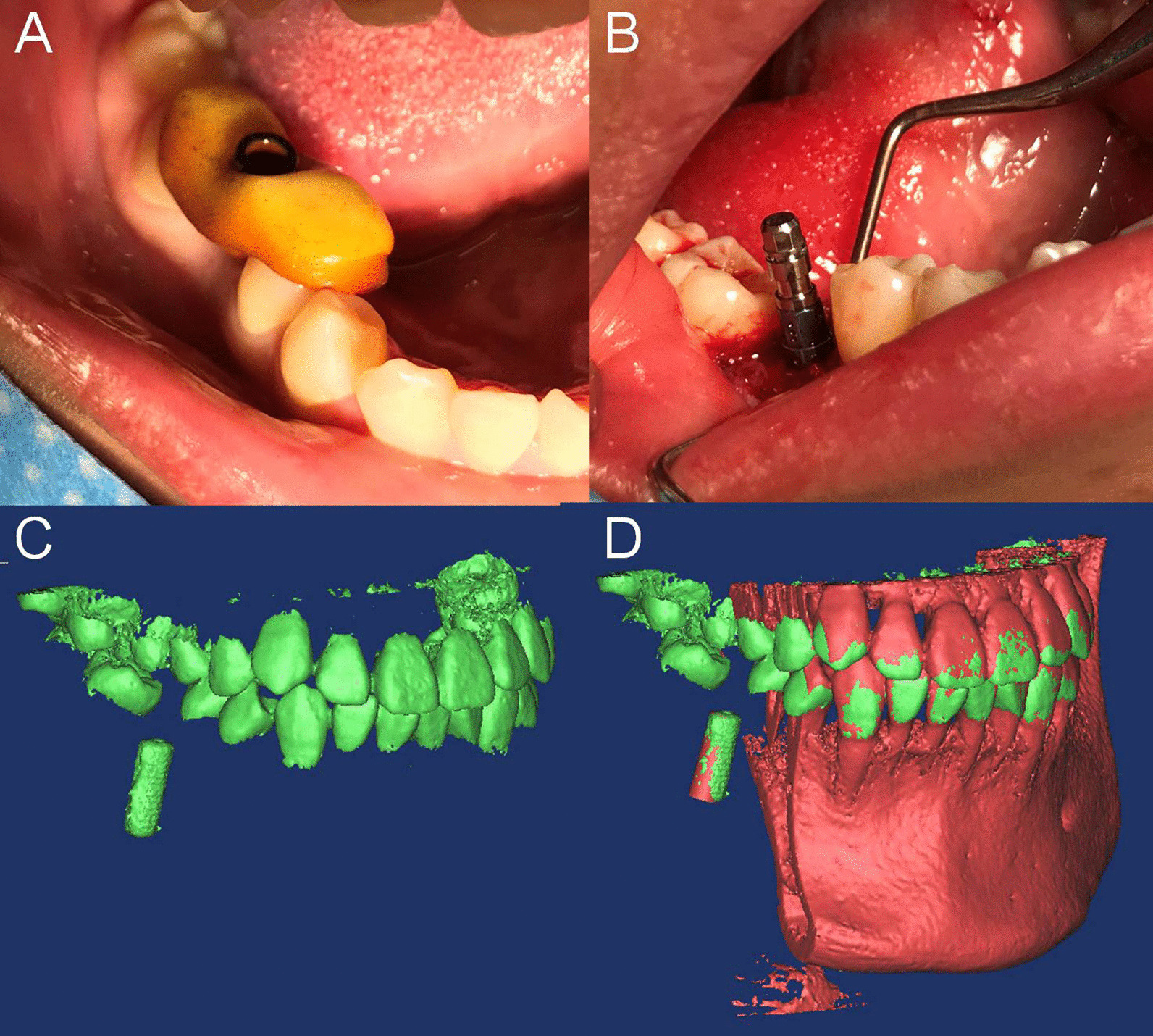
Guide implant placement and accuracy. **A** Inspection of the guide in place. **B** Placement of an implant (4.1 × 10 mm) for a missing right first mandibular molar. **C** Actual implant position. **D** Deviation validation between the virtual and actual implant positions

The guide was sterilized and inspected in place (Fig. 6A). After making an incision to create the flap, the 2.8-mm bar was guided by the TPT-based guide, and then preparation and implant placement were performed freehand. A bone lever implant (4.1 × 12 mm, BL, Straumann, Switzerland) was placed at the location of the right first mandibular molar (Fig. 6B). Postoperative CBCT was performed. Mimics 10.0 software was used to superimpose the postoperative implant position (Fig. 6C) with the virtual dental model used for preoperative planning (Fig. 6D). Differences in the position of the centre of the implant head and apex between preoperatively and postoperatively, as well as in the angular deviation of the implant axis, were calculated by the software. The total spatial deviation was obtained by calculating the differences between the planned and final positions on the x-, y-, and z-axes. Using the formula 3D deviation = √x^2^ + y^2^ + z^2^, a total spatial deviation value was obtained for this case that reflected the difference between the planned and postoperative implant positions. The central deviation of the implant head was 0.31 mm, the central deviation of the implant apex was 0.93 mm, and the implant angular deviation was 2.45°.

## Discussion and conclusions

At present, most implant guides rely on 3D scanning and 3D printing technology, and the accuracy is closely related to the precision of the guides [11]. The degree of registration of the scanned digital model and the CBCT model also has a great influence on the accuracy [12]. Some scholars have explored new techniques for creating implant guide plates, but the methods are more complicated [13]. TPT does not require 3D scanning, 3D printing, or registration, which can eliminate errors, and it can be directly applied using a plaster model, which has higher accuracy in terms of position. Nevertheless, TPT-based guide plate production may result in distortion with the use of alginate impression materials or light-cured temporary crown resin. In clinical processing, silicone rubber impression materials and low shrinkage resin materials can be used for guide plate processing, which can improve the accuracy.

The production of a guide plate by TPT can be divided into two main steps. First, because P1 is not well defined on the soft tissue of the missing tooth area, the position of P1 needs to be determined through the points P3 and P4 on the hard tissue of the tooth. Second, the position of P2 is determined by the positions of P1, P3, and P4 and the distance from three points to P2. The second step is achieved by two compasses and a fixed-length cylindrical bar.

TPT for guide creation is a low-cost process. After the implant position is designed by software, the operator only needs to obtain the coordinates and distances of four relevant points step by step to make the guide on the stone cast, which saves the time and cost of 3D printing. The procedure for making a guide by TPT is simple and programmable. After a doctor designs the implant position, the follow-up steps are only repeated mechanical operations, which could be completed by an assistant. Many CBCT software programs can simulate dental implants, and there is no need to use specific software to make a guide using TPT. Cylindrical guide bars, guide rings, compasses and digital callipers, etc., are not special equipment. Compared with the traditionally described computer-aided or s-CAIS methods for implant placement, considering differences among clinicians, whether this method for surgical guide development might be faster remains unknown. Differences in the processing time among these methods needs further study.

The indication for the tetrahedral localization technique is a single missing tooth or a few missing teeth. In cases of a single missing tooth, the form of retained tooth support can be used. The accuracy is related to the closeness of the guide plate to the support structure. Ozan et al. [14] evaluated the clinical application of 30 implant-supported dental guides and found an angular deviation of 2.91° ± 1.3° and head and tail deviations of 0.87 ± 0.40 mm and 0.95 ± 0.60 mm, respectively. Tahmaseb et al. [5] reviewed a total of 2238 implants placed in 471 patients using static guides. The spatial deviations recorded in the present study are smaller than those reported in the meta-analysis by Tahmaseb et al.: the total mean error was 1.2 mm at the entry point and 1.4 mm at the apical point, and the angular deviation was 3.5°, which is within the clinically acceptable range in the majority of clinical situations. Compared with conventional s-CAIS, a guide can be created by TPT directly using a solid model, which may improve the accuracy of the guide in place. Therefore, the deviations in the angle and length of the guide created by TPT were clinically acceptable. The accuracy of TPT for guide creation needs further research.

The reason why the distance between P1 and P2 was set to 39 mm is as follows. The cylindrical guide bar was designed to be 40 mm, from which 0.5 mm into the plaster model and 0.5 mm at the other end for fixing the compass tip needed to be subtracted. The closer the design is to a regular tetrahedron, the more stable its structure. The average width of the posterior segment of the maxillary and mandibular arch during normal occlusion (in males from southern China) is 49.20 mm and 36.19 mm, respectively [15]. Therefore, setting the length of the guide bar to 40 mm is beneficial for the clinical operation and stability of the guide.

The indication for guide creation by TPT is one missing tooth with adjacent tooth support or a few teeth missing, which is narrower than the indication for guide creation by 3D printing. The accuracy of TPT is affected by the subjectivity of the operator, the accuracy of the stone cast, the measuring instruments, and the shrinkage deformation of the curing materials. This workflow should be calibrated and tested with more than one operator in several cases. More studies with a larger number of patients are necessary to reach significant conclusions.

This case study illustrates the benefits of creating guides by tetrahedral positioning technology for the placement of dental implants. It is a new methodology for making implant guides that helps guide clinicians in locating the position of an implant to replace a single missing tooth. It is a promising technique offering a predictable outcome and lower risk of iatrogenic damage. Nonetheless, this should be interpreted with care since it is based on limited evidence from a case report. Additional studies are needed to validate the precision and reproducibility of this method.

## Data Availability

The complete data and materials described in the research article are freely available from the corresponding author on reasonable request.

## Abbreviations

TPT: Tetrahedron positioning technology
3D: Three dimensional
CBCT: Cone beam computer tomography
DICOM: Digital imaging and communications in medicine
CAD/CAM: Computer aided design/computer aided manufacturing
s-CAIS: Static computer-assisted implant surgery
CNC: Computer numerical control
